# Optimizing Eat, Sleep, Console in the NICU: a collaborative nursing education intervention

**DOI:** 10.1186/s40748-026-00277-4

**Published:** 2026-08-06

**Authors:** Sonia Kapoor, Caroline Chua, Laurie Reyna, Timothy M. Maul, Rochelle Sequeira Gomes

**Affiliations:** 1 Division of Neonatology, Nemours Children’s Health, Orlando, FL USA; 2 Division of Cardiothoracic Surgery, Nemours Children’s Health, Orlando, FL USA

**Keywords:** Neonatal opiate withdrawal syndrome (NOWS), Neonatal abstinence syndrome (NAS), Neonatology, Collaborative nursing education, Practice change, Family-centered care, Knowledge, attitudes and practices (KAP), Eat, Sleep and Console (ESC)

## Abstract

**Background:**

The Eat, Sleep, Console (ESC) model of care offers a patient-centered approach for managing neonatal opiate withdrawal syndrome (NOWS), reducing hospital stay, need for pharmacologic treatment, and costs compared to the traditional Finnegan scoring system. Favorable nursing knowledge, attitudes, and practices (KAP) are critical to successful ESC implementation. A prior pilot survey in our neonatal intensive care unit (NICU) revealed inconsistent understanding and application of ESC, indicating a need for targeted education. This study assesses changes in NICU nursing KAP scores following a three-month multiphase educational intervention, and identifies behavioral shifts, barriers, and opportunities to optimize ESC integration.

**Methods:**

We conducted a pre-post quasi-experimental study in a Level IV NICU using anonymous 11 point surveys completed by NICU nurses assessing knowledge, attitudes, and perceptions towards ESC before and after the collaborative educational intervention. The intervention included (1) a didactic session with open discussion, (2) an ESC poster displayed in the NICU, and (3) a one-page ESC reference guide. Statistical analyses utilized analysis of variance (ANOVA).

**Results:**

Thirty-three NICU nurses completed the pre- and post-intervention surveys; samples were independent and unmatched with an unknown degree of overlap. The increase in mean total KAP scores from 14.8 ± 2.2 to 15.3 ± 2.5 was not statistically significant (*p* = 0.40). Nurses with less than five years of experience showed the greatest improvement in knowledge and total KAP scores, increasing from 6.3 ± 1.5 to 8.3 ± 1.0 and from 14.3 ± 3.4 to 16.5 ± 1.9, respectively. Perceived safety and effectiveness of ESC improved slightly post-intervention. Didactic attendance did not significantly influence KAP (*p* > 0.80). Reported barriers to ESC implementation included staff and workflow constraints, limited family presence and parental involvement, lack of interdisciplinary support and system-level resources, and subjectivity and limitations of ESC assessments. Nurses emphasized the importance of greater social and family support, followed by increased nursing education, provider education, and improved lactation support.

**Conclusions:**

Collaborative education was not associated with statistically significant changes in NICU nursing KAP scores related to the ESC approach for NAS and may be insufficient as a stand-alone measure. Additional system-level support strategies may be needed to support ESC implementation fidelity and facilitate favorable nursing perceptions and practices towards ESC. Additionally, minimizing barriers affecting families and staff through tailored interventions may promote feasibility of ESC practices, also leading to improved nursing KAP.

**Supplementary Information:**

The online version contains supplementary material available at 10.1186/s40748-026-00277-4.

## Background

The Eat, Sleep, Console (ESC) model of care was originally described by Grossman et al. in 2014 for the management of infants with neonatal opioid withdrawal syndrome (NOWS), and has since been further standardized into a bedside care tool, as outlined by Young et al. [1, 2]. ESC has emerged as an alternative to the traditional Finnegan Neonatal Abstinence Scoring System (FNASS) and has increased in adoption due to its focus on non-pharmacological care and family involvement [1]. ESC focuses on functional assessment of the infant – can they eat, sleep and be consoled – based on defined thresholds for each function [1]. The ESC Care Tool standardizes the bedside assessment and guides interventions for symptomatic neonates [2]. It prioritizes the use of nonpharmacological consoling strategies first to manage symptoms, prior to giving as needed medication [1]. Benefits of this approach are decreased medication use, shorter length of hospital stay, and increased breastfeeding rates, without increased readmission rates or escalation of care, or adverse patient outcomes such as seizures [1, 2]. Of note, ESC outcomes have been primarily described for opioid withdrawal while data about other substances is limited [1, 2]. As ESC adoption expands, barriers such as resource limitations, competing staff priorities, knowledge deficits, and inconsistent parental involvement may compromise implementation fidelity [1, 3, 4].

NICU nurses play a vital role in clinical decisions in NAS management through their bedside care and assessments. Their perception and preferences may influence their knowledge, attitudes, and clinical practice in relation to the ESC approach. Since variation in nursing knowledge, attitudes, and practices (KAP) can lead to inconsistent application and integration of ESC, it is important to identify and then mitigate these barriers. Gallant et al.’s scoping review of 34 studies, primarily performed in the United States, revealed training and education was the most commonly used ESC implementation strategy (31.6%) [3].

Our Level IV NICU implemented ESC on July 1, 2024. While guidelines for ESC in our NICU exist, a prior pilot study revealed inconsistent understanding and application of ESC among nurses, indicating the need for additional targeted education and training. Although ESC outcomes in comparison to FNASS have been well-studied, there is limited research focused on the effect of collaborative education-based nursing interventions on nursing knowledge and perspectives following transition from FNASS to the ESC model of care. The primary objective of this study was to evaluate the effect of a three-month structured collaborative educational intervention on ESC on NICU nurses’ knowledge, attitude, and practice (KAP) scores using an anonymous survey. Secondary objectives assessed associations between years of experience and didactic attendance with KAP scores, and identified perceived barriers to and improvement opportunities for ESC implementation.

## Methods

### Study design and participants

This study employed a pre-post quasi-experimental design conducted in a 19 bed Level IV NICU using anonymous survey instruments with approval from the Institutional Review Board. Neonates at risk for or exhibiting symptoms of substance withdrawal after birth are referred to our NICU immediately or a few days after birth from their birthing hospitals. Our NICU has single family rooms with accommodations that facilitate rooming-in by two caregivers.

**Fig. 1:**
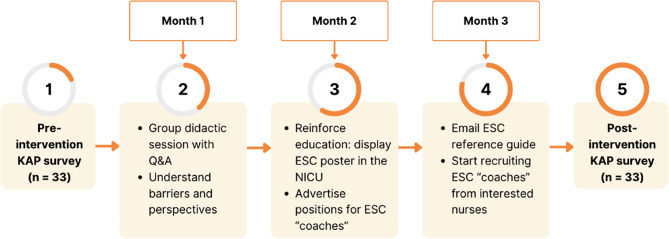
Study Flow Diagram and Timeline for NICU Nursing KAP Evaluation

Following completion of the pre-intervention study surveys, a 3-month multiphase intervention was implemented from August 2025 to October 2025. The intervention was based on the Eat, Sleep, Console model as described first by Grossman et al. [2]. The intervention included: [1] a group didactic session [2], display of an ESC poster in the NICU, and [3] distribution of ESC reference guides to nursing staff via email (Fig. 1).

#### Group didactic session

In month 1, a 60-minute group didactic session was conducted involving NICU nurses, the nursing educator, and physicians. The session included a review of ESC via a PowerPoint presentation, including benefits such as shorter length of stay and decreased need for medication. This was followed by a facilitated group discussion focused on nursing perspectives and perceived barriers to ESC implementation.

#### ESC poster

In month 2, an ESC poster (Supplement 1) reinforcing content from the initial didactic session in month 1 was displayed in the NICU, emphasizing how ESC assessments and treatments should be performed. It also included guidance on when a team huddle was indicated and how to use medication (morphine) when clinically indicated in the protocol.

#### ESC reference guide

In month 3, a one-page ESC reference guide was distributed to nursing staff via email to serve as an easy-to-use reference resource, particularly for float and support staff (Supplement 2). In addition, ESC “coaches” were recruited, but officially established following completion of the study period, to support future ESC integration.

Study population consisted of all NICU nurses currently working in our Level IV NICU. However, only individuals with bedside nursing certifications (RN, BSN) were included in the KAP surveys, while patient care technicians (PCTs), advanced practice providers (APRN, PA), physicians (DO, MD), and allied health professionals (RT, RD, PT, OT, SLP) were included in education but not in the surveys. Study participation was voluntary and anonymous, and no randomization, pre-post matching, or group assignments were performed.

### Sample size determination

Since the study population was from a single NICU and not all eligible NICU nurses were expected to participate, convenience sampling was used to obtain both the pre- and post-intervention surveys. Invitations were sent to all NICU nurses via email and survey QR codes were posted on unit huddle boards. With a significance level of 0.05 and 80% power, a moderate effect size (Cohen’s d = 0.50) would require approximately 64 participants.

### Data collection

We designed a structured questionnaire based on the KAP model. Both the pre- and post-surveys were advertised via posters displayed in the NICU and emails containing the survey QR-code and link. The pre-survey, administered to establish baseline data prior to any interventions, consisted of 11 items. The post-survey consisted of the same 11 items with the addition of a twelfth question evaluating the type and number of educational interventions individuals participated in. All survey questions were developed to reflect essential components of ESC and carefully revised for clarity prior to administration.

The survey evaluated four domains: professional experience (1 question), knowledge (6 questions), attitudes (2 questions), and practices (2 questions). All knowledge questions were either multiple-choice (single correct response) or checkbox-style (multiple correct responses). The attitudes and practices sections consisted of opinion-based questions utilizing Likert-scale, checkbox-style, and open-ended response formats. A structured multiple-response (checkbox) item assessed perceived strategies to improve ESC practices. Open-ended free text responses provided qualitative data on barriers to ESC implementation.

A scoring system was established to calculate both individual domain scores (K, A, and P) and total KAP scores. For multiple-choice items, 1 point was assigned for correct responses and 0 for incorrect. For checkbox-style and open-ended items, 1 point was given for each correctly selected or relevant response, respectively, up to the maximum number of correct options for that item. If incorrect or irrelevant answer options were present, no points were awarded or deducted for them. Three-point (0 = disagree, 1 = neutral, 2 = agree) and five-point (1 = strongly disagree, 2 = disagree, 3 = neutral, 4 = agree, 5 = strongly agree) Likert scales were used. Since the Likert scales differed in range, raw scores were calculated for each individually and then a single overall attitude score was created with both. The possible range of minimum and maximum scores for K, A, P, and total KAP was 0 to 10, 1 to 7, 1 to 7, and 2 to 24, respectively.

Open-ended responses were reviewed and grouped into thematic categories based on recurring ideas by a single investigator. Of note, responses could be assigned to more than one theme, if applicable. Frequencies accounted for overlapping themes and reported the number of responses reflective of each theme. The survey instrument and scoring framework are provided in Supplement 3.

### Data analysis

Data was scored using Microsoft Excel prior to statistical analysis in SPSS version 27. Assumptions of normality were assessed using the Kolmogorov-Smirnov test and visual inspection of Q-Q plots. Assumptions of homogeneity of variance were confirmed using Levene’s test. Descriptive statistics, such as mean, standard deviation, and frequency, summarized participant characteristics and survey responses. Data are presented in tables, figures, and graphs. Because the survey data could not be linked between the pre- and post-survey periods, comparisons of responses at the two time points were analyzed as two independent groups with time serving as a between-subjects factor. Statistical analyses utilized one-way analysis of variance (ANOVA) for time in each of the survey domains: knowledge (K), attitudes (A), practices (P), and total KAP. Additional comparisons were made for total KAP scores by didactic education attendance. Two-way ANOVA was performed to determine the main effects of survey period and years of NICU nursing experience, as well as their interaction, on domains and total KAP scores. All statistical testing was two-sided with alpha = 0.05.

## Results

### Professional experience

Thirty-three NICU nurses completed the pre- and post-intervention surveys to give a total of 66 survey results. The samples were independent and unmatched; therefore, the degree of overlap between the two groups is unknown. In the pre-survey, 4 (12.1%) had less than 5 years, 11 (33.3%) had 5 to 10 years, and 18 (54.6%) had greater than 10 years of NICU nursing experience. In comparison, the post-survey, 4 (12.1%) had less than 5 years, 8 (24.3%) had 5 to 10 years, and 21 (63.6%) had greater than 10 years of NICU nursing experience.

### Knowledge, attitudes, and practices

As shown in Table 1, no statistically significant improvement in mean total KAP scores was noted (14.8 ± 2.2 to 15.3 ± 2.5, *p* = 0.4) in the pre- to post-intervention setting. A two-way ANOVA revealed no significant main effect of years of NICU experience on individual or total KAP scores (*p* > 0.05). The greatest numerical increase in knowledge and total KAP scores was observed among nurses with less than five years of experience: 6.3 ± 1.5 to 8.3 ± 1.0 (*p* = 0.26) and 14.3 ± 3.4 to 16.5 ± 1.9 (*p* = 0.79), respectively. Additionally, didactic session attendance did not significantly influence individual or total KAP scores (*p* = 0.79).

**Table 1:** Comparison of pre- and post-intervention knowledge, attitude, and practice (KAP) scores among NICU nurses. Scores are stratified by years of NICU experience and attendance at the didactic session. Data are represented as mean ± standard deviation. P-values resulted from one-way^a^ and two-way^b^ ANOVA

Group	Pre-intervention Survey Score				Post-intervention Survey Score				*P*-value			
	K	A	*P*	KAP	K	A	*P*	KAP	K	A	*P*	KAP
All Nurses	6.6 ± 1.6	4.7 ± 1.1	3.5 ± 1.1	14.8 ± 2.2	6.9 ± 1.8	4.9 ± 1.1	3.4 ± 1.1	15.3 ± 2.5	0.51^a^	0.62^a^	0.56^a^	0.40^a^
Experience: < 5 years	6.3 ± 1.5	4.8 ± 1.0	3.3 ± 1.7	14.3 ± 3.4	8.3 ± 1.0	5.0 ± 1.2	3.3 ± 0.5	16.5 ± 1.9	0.26^b^	0.62^b^	0.87^b^	0.79^b^
Experience: 5–10 years	7.2 ± 1.5	4.6 ± 0.9	3.5 ± 0.8	15.4 ± 1.7	7.0 ± 1.9	4.6 ± 1.1	3.4 ± 1.3	15.0 ± 3.2				
Experience: > 10 years	6.3 ± 1.6	4.7 ± 1.2	3.6 ± 1.1	14.6 ± 2.1	6.6 ± 1.8	5.2 ± 1.1	3.4 ± 1.1	15.1 ± 2.3				
Didactic session: Yes					6.9 ± 1.9	4.7 ± 1.1	3.2 ± 1.1	14.8 ± 2.8	0.87^a^	0.62^a^	0.88^a^	0.79^a^
Didactic session: No					6.8 ± 1.8	5.3 ± 1.0	3.5 ± 1.0	15.7 ± 2.1				

^a^Between-group comparisons

^b^Effects of survey period and experience and their interaction

### Nursing attitudes towards ESC

**Fig. 2:**
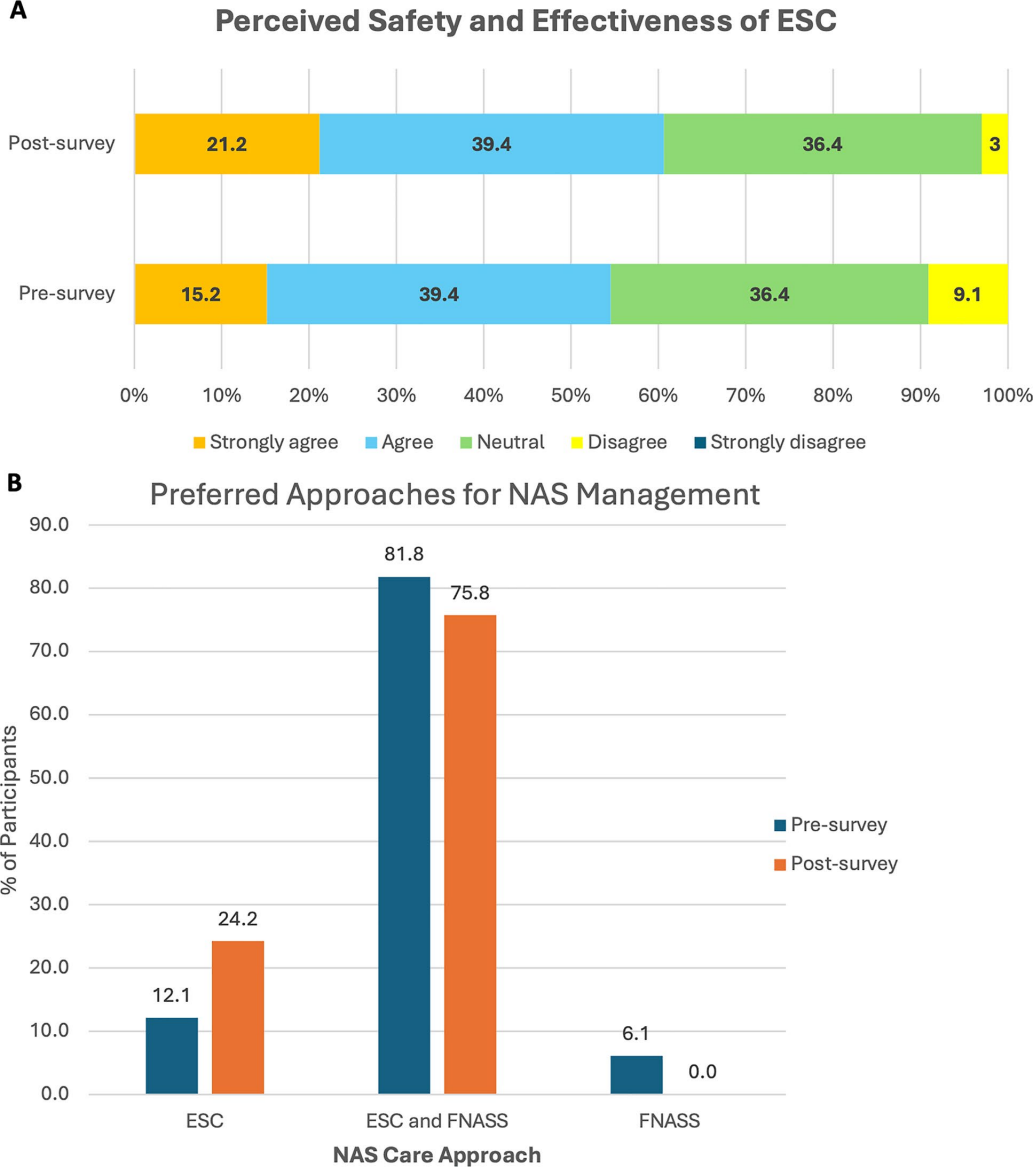
Changes in NICU Nurses’ Attitudes Regarding ESC After Educational Interventions. (**A**) Shift in Likert ratings, showing slightly stronger agreement with less disagreement. (**B**) Increased preference for ESC with a decrease for both ESC and FNASS or FNASS alone

Responses regarding perceived safety, effectiveness, and preference for ESC are described here descriptively and not analyzed for statistical significance. Post-intervention, a higher proportion of nurses reported greater perceived safety and efficacy of ESC. There was a 7% increase in “strongly agree” and a 6.1% decrease in “disagree” responses (Fig. 2A). Preference was also different. Fewer nurses selected FNASS (6.1% to 0%) and FNASS and ESC (81.8% to 75.8%) and more chose ESC (12.1% to 24.2%) in the post-intervention setting (Fig. 2B).

Qualitative feedback identified four main categories of barriers to effective ESC implementation (Table 2). The most frequently reported barrier was staffing and workflow constraints (*n* = 14), including inadequate staffing, competing patient assignments, and time constraints in a busy unit. Additional reported barriers were limited family presence and parental involvement (*n* = 13), lack of interdisciplinary support and system-level resources (*n* = 12), and subjectivity and limitations of ESC assessments (*n* = 3).

**Table 2:** Barriers to ESC implementation. Data was summarized from raw responses to identify common themes and frequencies of key barriers

Theme	*N*	Key Barriers
Staffing and workflow constraints	14	• Inadequate staffing • Time constraints • Acuity and/or number of patients assigned • Competing priorities • Difficult for staff to assist each other in a busy unit
Limited family presence and parental involvement	13	• Minimal and/or inconsistent parental involvement • Limited caregivers present to help console infant • Increases staff reliance to console infant
Lack of interdisciplinary support and system-level resources	12	• Limited staff and/or cuddlers to console infant • Need 1-to-1 care to consistently and effectively implement ESC • Lack of institutional investment in ESC • Minimal interdisciplinary teamwork
Subjectivity and limitations of ESC assessments	5	• Perceived subjectivity in scoring • ESC viewed as less comprehensive in comparison to FNASS • Variability depending on nurse experience

In terms of improvement strategies, the most frequently selected change to improve ESC practices was greater social and parental support (46%), followed by more nursing education (28%), more provider education (19%), and better lactation support (7%).

## Discussion

Our study showed that collaborative education did not significantly improve knowledge, attitude, and practice scores among NICU nurses. However, several important barriers to ESC implementation were identified such as staffing and workflow constraints, limited family presence and parental involvement, lack of interdisciplinary support and system-level resources, and subjectivity and limitations of ESC assessments. Notably, we observed improved perceptions of safety and preference towards ESC following the intervention. In our cohort, nurses with less than 5 years of experience were slightly more likely to demonstrate favorable KAP scores after the intervention compared to nurses with more than 5 years of experience.

Qualitative feedback identified limited parental presence at the bedside as a key barrier to ESC optimization. Caregivers play a critical role in non-pharmacological care of substance-exposed infants, including feeding and consoling, and contribute to infant regulation and bonding [5, 6]. Rooming-in has also been associated with decreased need for pharmacologic treatment and shorter hospital stays in comparison to detached care models [5, 6]. Therefore, improving caregiver presence has the potential to reduce nursing strains and minimize variability in ESC delivery [5].

Nurses also reported ongoing education as a supportive factor in improving ESC practices. In a referral Level IV NICU, inconsistent exposure to infants with NAS can limit opportunities for education, practice, and familiarity with ESC, potentially compromising standardization of care and confidence in its application and effectiveness.

System-level challenges within a referral Level IV NICU may further impact ESC fidelity. In comparison to birthing centers, establishing family engagement in the prenatal setting may be more challenging, hindering early integration of families into ESC-focused care [5, 6]. Efforts to actively welcome families to participate in care may help facilitate an inclusive, collaborative, and supportive environment while simultaneously reducing stigma associated with substance use [6]. These challenges are perpetuated by limited interdisciplinary support and staffing constraints, negatively impacting the feasibility of ESC integration and nursing perceptions of its clinical utility.

Given the overlapping barriers identified, a multifaceted approach may help mitigate these diverse challenges more effectively. For instance, prenatal guidance for families affected by substance use may be supported via telehealth when in-person communication is difficult, helping to establish expectations for the care of a substance-exposed newborn and prepare them for clinical engagement [5]. Additional potential strategies include an ESC care journal to encourage caregiver participation. For nursing staff, ongoing structured nursing education with feedback sessions and peer support from ESC coaches may reinforce consistent practice and improve nursing confidence [7]. While structured education and coaching likely strengthen knowledge directly, improving the feasibility of ESC implementation and addressing perceived barriers may also indirectly bolster knowledge and promote more favorable nursing attitudes and practices towards ESC.

Importantly, knowledge gaps remain in areas related to addiction and social determinants of health. One study revealed only 54% of nurses agreed or strongly agreed they had “enough knowledge about addiction to appropriately deal with mothers of infants with NAS” in comparison to 90% reporting adequate knowledge about NAS [7]. This highlights the need for targeted education addressing the complexities of addiction, stigma, and both implicit and explicit biases [7, 8]. Expanding educational content to include communication strategies with families, substance use stigma, and caregiver involvement may foster nursing knowledge, attitudes, and practice. By addressing caregiver, staff, and system-level barriers, these reinforcement strategies can create further opportunities for non-pharmacological interventions, strengthen nurse-caregiver partnerships, and improve both implementation fidelity of ESC and nursing KAP [7, 8].

The small positive shifts in perceived safety, effectiveness, and preference for ESC in our cohort were consistent with prior studies demonstrating high nurse satisfaction and strong support for parental involvement in infant care in NAS. At a regional medical center’s Level II NICU, 68% of nurses reported ESC was the best method for the management of NAS, and 97% felt ESC enhanced parental participation in infant care [9]. Similar to our findings, prior studies suggest newer nurses may be more receptive to evolving models of care, potentially reflecting fewer ingrained approaches to conventional management [10]. An important observation is that majority of nurses in our cohort reported a preference for both FNASS and ESC, likely reflecting an ongoing familiarity bias in support of the older FNASS system; however, our study did not adequately explore the aspects of this preference.

This study has several limitations, including limited generalizability, non-randomization, unpaired analysis, and the lack of longer-term assessment due to a small sample size and short intervention period. As a non-birthing center, there are fewer NAS admissions in comparison to NICUs with labor and delivery units, which may decrease opportunities for clinical application and reinforcement of ESC. Additionally, the KAP survey we utilized is an unvalidated tool and subjective, which may affect internal validity. The intervention primarily focused on the clinical aspects of ESC and did not comprehensively assess nursing KAP about other aspects of NAS care, such as communication and rapport with families, adverse social determinants of health, and substance use-related stigma. These factors may influence KAP and reflect prior experience with the FNASS model. Finally, while our ESC approach was primarily based on the ESC model described by Grossman et al., using the more widely studied ESC Care Tool may facilitate further standardization, robust implementation, and education opportunities, such as case-based simulations and trauma-informed care [1, 2].

Future work will focus on establishing sustainable support systems to enhance ESC practices among NICU nurses. Current priorities include the active integration of ESC coaches and dedicated infant cuddlers, routine opportunities for nursing feedback, and interdisciplinary collaboration with lactation consultants and social workers. A longer follow-up period will allow evaluation of the impact of these strategies over time. Future studies will incorporate paired statistical analysis to improve power and decrease variability. Furthermore, KAP surveys will be broadened to assess social domains of NAS care, providing a more comprehensive understanding of factors capable of influencing ESC implementation.

## Conclusions

Collaborative education was not associated with statistically significant changes in nursing KAP related to the ESC approach and may be insufficient as a stand-alone strategy. Effective and sustained ESC adoption and favorable staff attitudes may require additional support strategies, further exploration of perceptions and biases, and ongoing educational reinforcements. Recognizing and addressing barriers at the family, staff, and system levels may help design inclusive, bidirectional, and collaborative improvement strategies that could promote consistent practice, support family-centered care, and optimize patient outcomes, while also indirectly enhancing nursing KAP towards ESC.

## Supplementary Information

Below is the link to the electronic supplementary material.

Supplementary Material 1

Supplementary Material 2

Supplementary Material 3

## Data Availability

The datasets generated during the current study are available from the corresponding author on reasonable request.

## Abbreviations

NOWS: Neonatal opiate withdrawal syndrome
NAS: Neonatal abstinence syndrome
ESC: Eat, Sleep, Console
FNASS: Finnegan Neonatal Abstinence Scoring System
KAP: Knowledges, attitudes, and practices
NICU: Neonatal intensive care unit
PCT: Patient care technician
