# Supplementary material for: Optimizing Eat, Sleep, Console in the NICU: a collaborative nursing education intervention
Source: Matern Health Neonatol Perinatol. 2026 Aug 6;12:34. doi: 10.1186/s40748-026-00277-4 (PMC13445891; doi:10.1186/s40748-026-00277-4)
Supplement: Supplementary file 1 [file 40748_2026_277_MOESM1_ESM.pdf]

Eat, Sleep, Console (ESC): Management of Neonatal Abstinence Syndrome (NAS)

What is ESC?

- ESC is a novel approach to assess infants with substance withdrawal
  - Implemented at NCH NICU (Orlando, FL) in 2024
- Finnegan NAS tool traditionally focuses on the number and severity of symptoms to determine pharmacological management, while ESC prioritizes non-pharmacological care and family involvement to reduce exposure to medication and length of hospital stay

Why is ESC preferred?

- Less disruptive assessment for infants
- Reduced time until medically ready for discharge by 6.7 days
- Shorter length of hospital stay by 6.2 days
- Decreased medication use by 32.5%
- Encourages caregiver presence and involvement

*Based on ESC-NOW trial at 26 U.S. sites

How to use ESC?

- Perform every 3 – 4 hours with routine infant care
- Incorporate input from all caregivers
- Document on the ESC flowsheet in EPIC
- Continue for:
  - At least 48 hours for short-acting opioids
  - 4 – 7 days for long-acting opioids
  - 24 – 48 hours after stopping pharmacological treatment

Eat

Does the infant have poor eating due to NAS?

- Goal:
  - At least 1 ounce per feed
  - Coordinate feeding within 10 minutes of hunger cues
  - Feeding for at least 10 minutes
- *For premature infants, establish baseline based on feeding patterns for their gestational and postnatal age*

Sleep

Did the infant sleep less than 1 hour after feeding due to NAS?

- Goal:
  - Sleep at least 1 hour with or without consoling interventions
- *Sleep < 1 hour is appropriate if it is due to physiological cluster feeding*

Console

Is the infant inconsolable within 10 minutes due to NAS?

- Goal:
  - Consolable within 10 minutes with or without consoling support interventions (CSIs)

Scoring level of support:

1. **Soothes with little support** (any of the first 4 CSIs): calm voice, hand-to-mouth, hand on abdomen, arms and legs to center
2. **Soothes with some support**: skin-to-skin, swaddling, feeding, sucking on pacifier or finger, rocking/swaying
3. **Soothes with much support** or does not soothe in 10 minutes

Pharmacological Management

- 1st line: oral morphine (0.4 mg/ml)
  - Starting dose: 0.05 mg/kg/dose every 3 hours **as needed**
  - Escalate to scheduled if needed
- 2nd line: phenobarbital

Team Huddle

- Recommended if **"Yes"** response to any ESC item or infant receives scores of **"3"** for soothing support used
- Involves bedside RN, infant's parent, physician or associate provider, and, if possible, social worker
- Discuss optimizing non-pharmacological care and feeding, caregiver presence, infant's environment, and efficacy of CSIs

Sources

- Grossman, M. R., Lipshaw, M. J., Osborn, R. R., & Berkwitt, A. K. (2018). A Novel Approach to Assessing Infants With Neonatal Abstinence Syndrome. *Hospital Pediatrics*, *8*(1), 1–6. https://doi.org/10.1542/hpeds.2017-0128
- Singh, R., & Davis, J. M. (2021). Escaping the Finnegan - Is it time? *Seminars in Fetal and Neonatal Medicine*, *26*(3). https://doi.org/10.1016/j.siny.2021.101218
- Young, L. W., Ounpraseuth, S. T., Merhar, S. L., Hu, Z., Simon, A. E., Bremer, A. A., Lee, J. Y., Das, A., Crawford, M. M., Greenberg, R. G., Smith, P. B., Poindexter, B. B., Higgins, R. D., Walsh, M. C., Rice, W., Paul, D. A., Maxwell, J. R., Telang, S., Fung, C. M., ... Devlin, L. A. (2023). Eat, Sleep, Console Approach or Usual Care for Neonatal Opioid Withdrawal. *New England Journal of Medicine*, *388*(25), 2326–2337. https://doi.org/10.1056/nejmoa2214470
- Chua, C., & Tillery, R. (2025). *NICU Survival Manual* (2nd ed.). Nemours Children's Hospital
