# Supplementary material for: Optimizing Eat, Sleep, Console in the NICU: a collaborative nursing education intervention
Source: Matern Health Neonatol Perinatol. 2026 Aug 6;12:34. doi: 10.1186/s40748-026-00277-4 (PMC13445891; doi:10.1186/s40748-026-00277-4)
Supplement: Supplementary file 2 [file 40748_2026_277_MOESM2_ESM.pdf]

Eat, Sleep, Console (ESC) – A Quick Reference Guide

1. ESC Assessment

- Perform every 3-4 hours with infant care
- Incorporate input from all caregivers
- Document findings on the ESC flowsheet in EPIC
- Continue for:
  - At least 48 hours – short-acting opioids
  - 4 – 7 days – long-acting opioids
  - 24-48 hours after medications stop

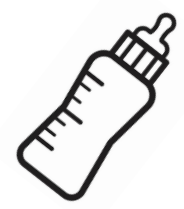<br>**Eat**	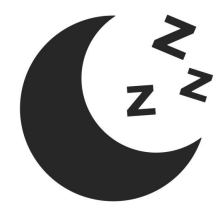<br>**Sleep**	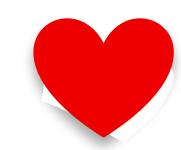<br>**Console**
Does the infant have poor eating due to NAS – ***Yes or No?***	Did the infant sleep < 1 hour after feeding due to NAS – ***Yes or No?***	Is the infant inconsolable within 10 minutes due to NAS – ***Yes or No?***
Goal:<br>• ≥ 1 oz per feed<br>• Coordinate feeding within 10 minutes of hunger cues<br>• Feeding for ≥ 10 minutes	Goal:<br>• ≥ 1 hour with or without consoling interventions	Goal:<br>• Consolable within 10 minutes with or without CSIs

2. Non-Pharmacologic Care: Consoling Support Interventions (CSIs)

1. Soothes with little support.
   - Calm voice, hand-to-mouth, hand on abdomen, and arms and legs to center
2. Soothes with some support.
   - Skin-to-skin, swaddling, feeding, sucking on a pacifier or finger, and rocking/swaying
3. Soothes with much support or does not soothe in 10 minutes.

3. When to Initiate a Team Huddle

- If there is a **"Yes"** response to any ESC item or infant receives scores of **"3"** for CSI score
- Involves at least the bedside RN and infant's parent along with physician or associate provider and social worker
- Opportunity to discuss optimization of non-pharmacological care and feeding, caregiver presence, infant's environment, and efficacy of CSIs

4. Pharmacological Therapy

- 1st line: Oral Morphine (0.4 mg/ml) → starting dose: 0.05 mg/kg/dose every 3 hours PRN
  - Escalate to scheduled if necessary
- 2nd line: Phenobarbital → initiate when Morphine administration reaches a 1.2 mg/kg/day, morphine is unable to weaned by day 7 of treatment, and/or there is concern for polysubstance withdrawal
