# Supplementary material for: Optimizing Eat, Sleep, Console in the NICU: a collaborative nursing education intervention
Source: Matern Health Neonatol Perinatol. 2026 Aug 6;12:34. doi: 10.1186/s40748-026-00277-4 (PMC13445891; doi:10.1186/s40748-026-00277-4)
Supplement: Supplementary file 3 [file 40748_2026_277_MOESM3_ESM.pdf]

Eat Sleep Console – A Nursing Survey of Knowledge, Attitudes, and Practices

Thank you for your participation in this educational intervention to optimize our Eat sleep console knowledge, attitudes and practices. Please know that this survey is voluntary and anonymous. No identifier information is requested or tracked.

1. I have ___ years of NICU nursing experience
   a. < 5
   b. 5 - 10
   c. >10

   Knowledge

2. Select all the factors that signify a baby can eat "well."
   a. Eats ≥ 1 oz every 2-3 hours
   b. Eats on a fixed schedule
   c. Eats on demand
   d. Without excessive gagging or drooling

3. After feeding, a baby, who is monitored for withdrawal, wakes up crying multiple times during his 2 hour nap. This baby would score a "No" for sleep in Eat, sleep, Console.
   a. True
   b. False

4. A baby soothes easily with skin-to-skin contact, swaddling, rocking or swaying, sucking on a pacifier, or feeding. This baby
   a. Soothes with little support (1)
   b. Soothes with some support (2)
   c. Soothes with much support or does not soothe in 10 minutes (3)

5. A neonate with in utero exposure to heroin is eating ½ oz per feed, sleeping for 45 minutes, and is inconsolable. Select the next best step(s)

a. Optimize consoling strategies
b. Team huddle
c. Trial of scheduled enteral morphine
d. Draw blood culture and start antibiotics

6. In comparison to the Finnegan Neonatal Abstinence Scoring System (FNASS), infants assessed and managed with Eat, Sleep, Console (ESC) have
   a. Longer hospital length of stay
   b. Decreased pharmacological treatment
   c. Fewer NICU admissions

7. Family involvement in the care of NAS babies has been shown to decrease length of hospital stay, need for medication, and severity of withdrawal symptoms.
   a. True
   b. False

Attitudes

8. ESC is a safe and effective method for managing neonatal abstinence syndrome.
   a. Strongly disagree
   b. Disagree
   c. Neutral
   d. Agree
   e. Strongly agree

9. In my opinion, the most effective approach(es) to care for a baby with NAS is/are
   a. Only ESC scoring
   b. ESC and Finnegan scoring
   c. Only Finnegan scoring

Practices

10. In my experience, barriers to effectively implementing ESC are ___________________.

11. In my opinion, to improve our ESC practices, the following changes would be helpful
   a. More nursing education
   b. More provider education
   c. Greater social and parental support
   d. Better lactation support

Follow up survey only

12. Please mark educational interventions and resources you have used/ attended in the last three months
   a. ESC presentation at Beyond the Incubator
   b. Email with easy-to-use ESC guide

c. ESC poster
d. ESC coach

Question #	Category	Scoring Method	Answer	Minimum Score	Maximum Score
1	N/A	Free response	A, B, or C	0	0
2	Knowledge	Each correct answer is 1 point. Each incorrect answer is 0 points.	A, C, and D	0	3
3	Knowledge	Correct: 1 point<br>Incorrect: 0 points	B	0	1
4	Knowledge	Correct: 1 point<br>Incorrect: 0 points	B	0	1
5	Knowledge	Each correct answer is 1 point. Each incorrect answer is 0 points.	A and B	0	2
6	Knowledge	Each correct answer is 1 point. Each incorrect answer is 0 points.	B and C	0	2
7	Knowledge	Correct: 1 point<br>Incorrect: 0 points	A	0	1
8	Attitudes	Strongly disagree: 1<br>Disagree: 2<br>Neutral: 3<br>Agree: 4<br>Strongly Agree: 5	Opinion	1	5
9	Attitudes	Only Finnegan scoring = Disagree: 0<br>ESC and Finnegan scoring = Neutral: 1<br>Only ESC scoring = Agree: 2	Opinion	0	2
10	Practices	Free response. 1 point (up to a maximum of 3) for each of the following mentioned to the right.<br>1 point – 1 barrier<br>2 points – 2 barriers	Can include the following: education/training, resistance to change, physician preference, challenges that decrease parental/guardian	0	3

		3 points – at least 3 barriers	involvement, neonatal co-morbidities.		
11	Practices	Each correct answer is 1 point. Each incorrect answer is 0 points.	A, B, C, and D	1	4
12	N/A	Free response. Each selected option is assigned 1 point. Additionally, mark 1 for attendance and 0 for absence at ESC presentation.	A, B, C, and/or D	0	4

Range of Composite KAP Scores:

- Knowledge: 0 - 10
- Attitudes: 1 – 7
- Practices: 1 – 7
- Total: 2 – 24
